# Supplementary material for: Health care expenditure in the last five years of life is driven by morbidity, not age: A national study of spending trajectories in Danish decedents over age 65
Source: PLoS One. 2020 Dec 18;15(12):e0244061. doi: 10.1371/journal.pone.0244061 (PMC7748135; doi:10.1371/journal.pone.0244061)
Supplement: S1 Table — (DOCX) [file pone.0244061.s002.docx]

### S1 Table. Grouping of causes of death – definition and numbers

| Cause of death | n | % | ICD-10 codes |
| --- | --- | --- | --- |
| Cancer | 61475 | 28·2 | C00-97 |
| Heart diseases | 36394 | 16·7 | I00-25, I27, I30-51 |
| Diseases of the respiratory system | 27705 | 12·7 | J00-99 |
| Other cardiovascular diseases | 20337 | 9·3 | I26, I28, I60-99 |
| Dementia | 18622 | 8·5 | F00, F01, F020, F03, G30, G318, G319 |
| Diseases of the digestive system | 7674 | 3·5 | K00-92 |
| Symptoms and abnormal findings, ill-defined causes | 7433 | 3·4 | R00-98, R999 |
| Endocrine, nutritional and metabolic diseases | 7181 | 3·3 | E00-90 |
| Deaths with no medical information | 6726 | 3·1 | R990 |
| Accidents | 4443 | 2 | V01-X59, Y40-69,Y70-86, Y88 |
| Diseases of the nervous system and sensory organs | 4310 | 2 | G00-29, G310-7, G35-H95 |
| Infectious and parasitic diseases | 4296 | 2 | A00-B99 |
| Diseases of the genitourinary system | 4052 | 1·9 | N00-98 |
| Mental and behavioural disorders | 2011 | 0·9 | F04-99 |
| Diseases of the musculoskeletal system and connective tissue | 1657 | 0·8 | M00-99 |
| Other neoplasms | 1407 | 0·6 | D00-48 |
| Diseases of the blood and blood-forming organs | 1061 | 0·5 | D50-89 |
| Suicide and attempted suicide | 918 | 0·4 | X60-84, Y870 |
| Diseases of the skin and subcutaneous tissue | 279 | 0·1 | L00-99 |
| Congenital malformations, and chromosomal anomalies | 176 | 0·1 | Q00-99 |
| Events of undetermined intent | 40 | 0 | Y10-34, Y872, Y899 |
| Assault | 24 | 0 | X85-99, Y00-09, Y871 |
| Certain conditions originating in the perinatal period | 10 | 0 | P00-96 |
