# Supplementary material for: Health care expenditure in the last five years of life is driven by morbidity, not age: A national study of spending trajectories in Danish decedents over age 65
Source: PLoS One. 2020 Dec 18;15(12):e0244061. doi: 10.1371/journal.pone.0244061 (PMC7748135; doi:10.1371/journal.pone.0244061)
Supplement: S2 Table — (DOCX) [file pone.0244061.s003.docx]

**S2 Table. Healthcare expenditure (per-person 1000€) in the last five years of life, and proportion of hospital costs by age at death and latent class membership**

|  | Low Expenditures | | Accelerating Expenditures | | Moderate Persistent Expenditures | | High Persistent Expenditures | |
| --- | --- | --- | --- | --- | --- | --- | --- | --- |
|  | mean | (IQR) | mean | (IQR) | mean | (IQR) | mean | (IQR) |
| Healthcare expenditure by age at death |  |  |  |  |  |  |  |  |
| 65-74 | 16 | (0-22) | 52 | (17-73) | 64 | (28-87) | 152 | (87-184) |
| 75-84 | 13 | (0-16) | 41 | (14-58) | 55 | (28-74) | 131 | (79-155) |
| 85-94 | 9 | (0-13) | 34 | (13-47) | 53 | (29-73) | 121 | (79-142) |
| 95+ | 6 | (0-6) | 37 | (25-54) | 61 | (37-84) | 125 | (82-144) |
| Proportion hospital costs by age at death |  |  |  |  |  |  |  |  |
| 65-74 | 59 | (0-97) | 82 | 81-97 | 76 | 68-94 | 71 | (56-93) |
| 75-84 | 50 | (0-95) | 74 | 64-94 | 68 | 53-89 | 58 | (33-84) |
| 85-94 | 39 | (0-89) | 61 | 38-88 | 52 | 26-78 | 39 | (17-58) |
| 95+ | 22 | (0-44) | 45 | 15-74 | 32 | 8-53 | 25 | (9-35) |
