# Supplementary material for: Health care expenditure in the last five years of life is driven by morbidity, not age: A national study of spending trajectories in Danish decedents over age 65
Source: PLoS One. 2020 Dec 18;15(12):e0244061. doi: 10.1371/journal.pone.0244061 (PMC7748135; doi:10.1371/journal.pone.0244061)

## S1 Appendix: Further description of the latent trajectory analysis

### Fit statistics for the latent class models considered

| Degree of polynomial | No. of classes | BIC | Entropy | Percent in each class based on most likely class membership |
| --- | --- | --- | --- | --- |
| 1 | 1 | 2107249 | 1 | 100 |
| 2 |  | 2106846 | 1 | 100 |
| 3 |  | 2106763 | 1 | 100 |
| 1 | 2 | 1967787 | 0·98 | 14 / 86 |
| 2 |  | 1966252 | 0·98 | 14 / 86 |
| 3 |  | 1966127 | 0·98 | 14 / 86 |
| 1 | 3 | 1913053 | 0·92 | 6 / 34 / 60 |
| 2 |  | 1910803 | 0·92 | 6 / 34 / 60 |
| 3 |  | 1910635 | 0·92 | 6 / 34 / 60 |
| 1 | 4 | 1889549 | 0·9 | 3 / 43 / 43 / 10 |
| 2 |  | 1886910 | 0·91 | 3 / 43 / 10 / 43 |
| 3 |  | 1886747 | 0·91 | 3 / 10 / 43 / 43 |
| 1 | 5 | 1876415 | 0·9 | 4 / 11 / 5 / 46 / 34 |
| 2 |  | 1873717 | 0·9 | 5 / 11 / 46 / 35 / 4 |
| 3 |  | 1872914 | 0·9 | 4 / 34 / 5 / 46 / 12 |
| 1 | 6 | 1865628 | 0·89 | 2 / 47 / 5 / 5 / 17 / 25 |
| 2 |  | 1862511 | 0·9 | 2 / 5 / 17 / 46 / 5 / 25 |
| 3 |  | 1861223 | 0·9 | 2 / 5 / 5 / 17 / 46 / 25 |
| 1 | 7 | 1859031 | 0·88 | 2 / 42 / 16 / 9 / 4 / 4 / 23 |
| 2 |  | 1855221 | 0·9 | 2 / 3 / 6 / 4 / 18 / 45 / 23 |
| 3 |  | 1854278 | 0·9 | 2 / 17 / 3 / 44 / 6 / 4 / 23 |
| 1 | 8 | 1853684 | 0·88 | 6 / 42 / 17 / 8 / 20 / 3 / 3 / 1 |
| 2 |  | 1849519 | 0·89 | 1 / 2 / 5 / 3 / 5 / 43 / 22 / 18 |
| 3 |  | 1848516 | 0·89 | 1 / 5 / 2 / 3 / 21 / 43 / 6 / 18 |

### Parameter estimates for the final latent trajectory model

|  |  |  | Estimate |  | Standard  error |
| --- | --- | --- | --- | --- | --- |
| Class 1 | |  |  |  |  |
|  | Intercept |  | -4·32 |  | 0·022 |
|  | Dummy, last quarter of life |  | 3·61 |  | 0·031 |
|  | Time |  | 0·94 |  | 0·017 |
|  | Time^2^ |  | 0·15 |  | 0·003 |
| Class 2 | |  |  |  |  |
|  | Intercept |  | 1·80 |  | 0·013 |
|  | Dummy, last quarter of life |  | 2·03 |  | 0·018 |
|  | Time |  | 2·04 |  | 0·011 |
|  | Time^2^ |  | 0·24 |  | 0·002 |
| Class 3 | |  |  |  |  |
|  | Intercept |  | 2·17 |  | 0·006 |
|  | Dummy, last quarter of life |  | 2·03 |  | 0·009 |
|  | Time |  | 0·65 |  | 0·005 |
|  | Time^2^ |  | 0·07 |  | 0·001 |
| Class 4 | |  |  |  |  |
|  | Intercept |  | 3·20 |  | 0·004 |
|  | Dummy, last quarter of life |  | 1·48 |  | 0·008 |
|  | Time |  | 0·05 |  | 0·004 |
|  | Time^2^ |  | -0·03 |  | 0·001 |

Mean, log-scale trajectories of the classes of the alternative latent trajectory models


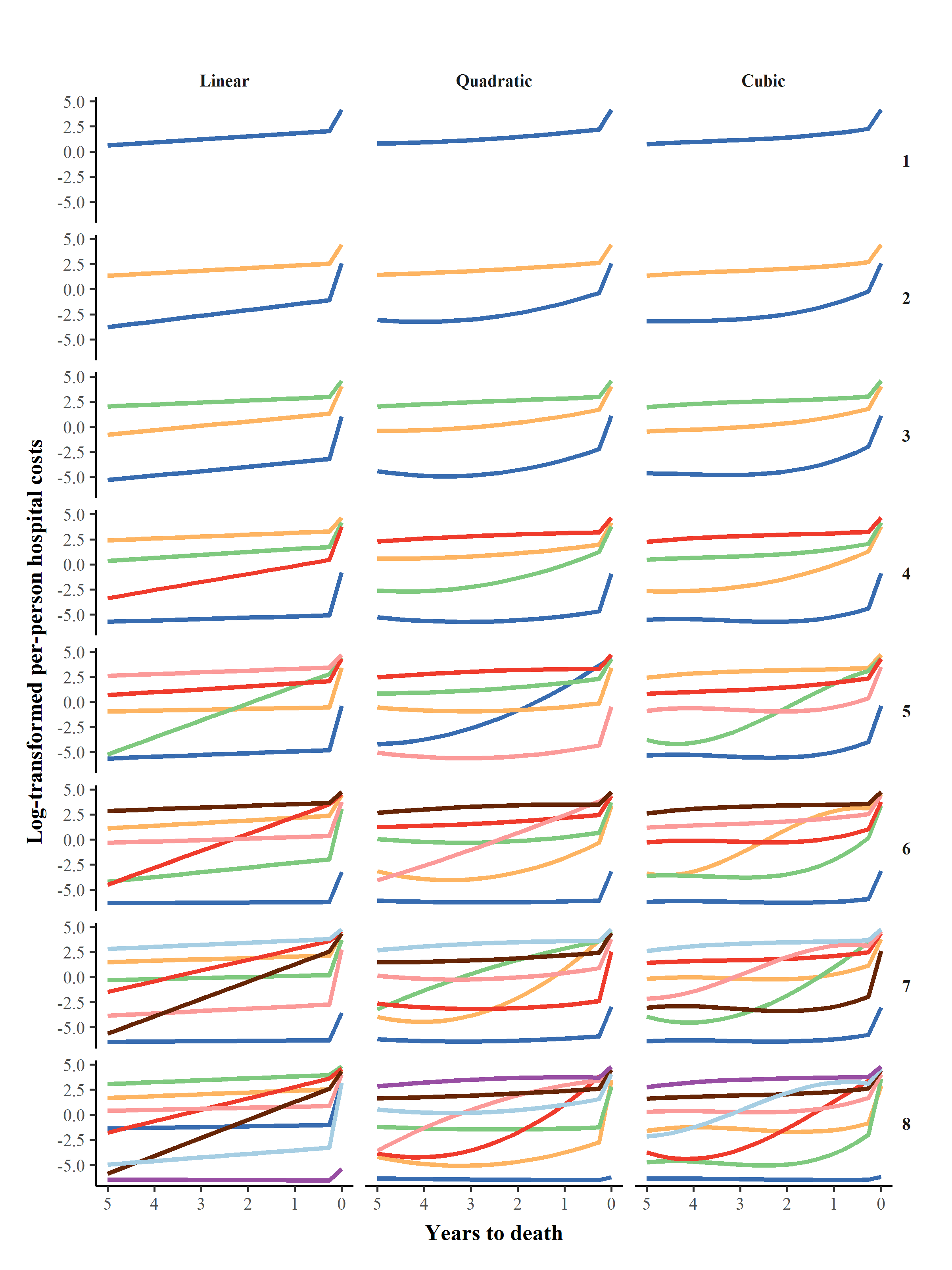

Supplement: S1 Appendix — (DOCX) [file pone.0244061.s004.docx]
